# Expression of the Wnt ligands gene family and its relationship to prognosis in hepatocellular carcinoma

**DOI:** 10.1186/s12935-019-0743-z

**Published:** 2019-02-15

**Authors:** Jia-Jia Dong, Li Ying, Ke-Qing Shi

**Affiliations:** 1 Department of Ultrasonography, the First Affiliated Hospital of Wenzhou Medical University, Wenzhou, Zhejiang China; 2 Precision Medical Center, First Affiliated Hospital of Wenzhou Medical University, Wenzhou, China; 3 Key Laboratory of Diagnosis and Treatment of Severe Hepato-Pancreatic Diseases of Zhejiang Province, Wenzhou, China

**Keywords:** Wnt ligands, TCGA, Prognosis, Biomarker, Hepatocellular carcinoma

## Abstract

**Background:**

The Wnt gene family members are known to participate regulating various normal and pathological processes including tumorigenesis. However, the association between Wnt ligands gene family and prognosis in hepatocellular carcinoma has not been systematically studied. Therefore, we evaluated the role of Wnt ligands gene family in hepatocellular carcinoma using publicly available data from The Cancer Genome Atlas (TCGA).

**Methods:**

Clinical information and RNA-Seq mRNA expression data were derived from TCGA hepatocellular carcinoma cohort. Differences in overall survival (OS) and disease-free survival (DFS) between increased and decreased expression groups (defined by X-tile analyses) of Wnt ligands gene family were compared using Kaplan–Meier method and Cox regression model, with p-values calculated via log-rank test. Gene Set Enrichment Analysis (GSEA) was performed.

**Results:**

Multivariate analysis adjusted for patient age, sex, BMI, tumor grade, and TMN stage revealed that Wnt1, Wnt3 and Wnt5B expressions were independent prognostic factors for OS and DFS (OS: HR = 0.58, P = 0.006; HR = 0.65, P = 0.03; HR = 0.56, P = 0.023, respectively; DFS: HR = 0.52, P < 0.001; HR = 1.93, P = 0.003; HR = 0.59, P = 0.011, respectively). Furthermore, expression of Wnt1 and Wnt5B was significantly associated with TMN stage (P = 0.02 and P = 0.03 for OS; P = 0.02 and P = 0.02 for DFS). GSEA showed that nucleotide excision repair was differentially enriched in Wnt1 low expression phenotype and aminoacyl trna biosynthesis and basal transcription factors were differentially enriched in Wnt5B low expression phenotype.

**Conclusions:**

Our results identified associations of several Wnt ligands with prognosis of HCC patients, indicating that these genes could serve as prognostic biomarkers of HCC.

**Electronic supplementary material:**

The online version of this article (10.1186/s12935-019-0743-z) contains supplementary material, which is available to authorized users.

## Background

Liver cancer is one of the most commonly diagnosed cancer and the second most frequent cause of cancer-related deaths globally, with 854,000 incident cases and 810,000 deaths in 2015 [1]. Hepatocellular carcinoma (HCC), as the most common pathological type, accounts for approximately 90% of primary liver cancers [2]. Chronic viral hepatitis (B and C), alcohol intake and aflatoxin exposure were well known underlying aetiologies. A series of therapies including liver resection, percutaneous ethanol injection, transcatheter arterial chemoembolization, microwave ablation, liver transplantation and systemic therapy were developed for patients with HCC. However, the mortality rate of HCC was always very high due to their disease being diagnosed at a late stage [3]. Detailed underlying mechanisms of development and progression of HCC were considered complicated and ambiguous. Studying the genes that play a key role in HCC development is crucial to identify disease biomarkers which could be of great use for diagnosis, prognostic prediction or even development of targeted drugs.

The Wnt family of 19 secreted glycoproteins have crucial roles in the regulation of diverse processes, such as embryogenesis, differentiation, and tumorigenesis through canonical dependent and non-canonical pathways [4, 5]. Numerous studies have reported that aberrant activation of Wnt signaling may contribute to the pathology of various types of cancer, including colon cancer [6], gastric cancer [7], HCC [8]. A previous study, using glutamine synthetase (encoded by canonical Wnt signaling target GLUL gene) as a sensitive and specific marker, showed that 36% HCCs displayed canonical Wnt activation [9]. Recently, Wnt3a expression in HCC is reported to be associated with the poorly-differentiated grade, liver cirrhosis, HBV infection, higher TNM stage, and a relatively shorter survival time [10]. Moreover, several previous investigations demonstrated that an increased Wnt1 expression was detected in human HCC tissue and human hepatoma cell lines and correlated with increased tumor recurrence after curative tumor resection [11, 12]. In addition to Wnt3a and Wnt1, increased expression of Wnt3, Wnt4, Wnt5a, and Wnt10b has also been revealed in HCC tumors and in the peritumoral liver tissues [8, 13, 14]. However, a comprehensive analysis of the association between the expression of Wnt ligands and clinicopathologic features of hepatocellular carcinoma (HCC) is lacking. Thus, the objective of the current study was to extensively evaluate the prognostic value of Wnt ligands in HCC based on data obtained from TCGA. To gain further insight into the biological pathways involved in HCC pathogenesis related Wnt regulatory network, GSEA was also performed.

## Materials and methods

The level-3 expression data (RNA-seqV2) and clinicopathological data of 360 HCC patients and 50adjacent normal liver samples were downloaded from The Cancer Genome Atlas (TCGA, https://tcga-data.nci.nih.gov/tcga/) data portal. The clinicopathological characteristics of HCC patients, including age, sex, race, body mass index (BMI), tumor grade, tumor, node, metastasis (TNM) stage, overall survival time, overall survival status, disease free survival time and disease-free survival status, were collected. The methods of biospecimen collection, RNA isolation, and RNA sequencing were previously described by the Cancer Genome Atlas Research Network [15]. The data was processed according to the TCGA publication guidelines and data access policies.

### Gene set enrichment analysis

To determine whether a priori defined set of genes shows statistically significant, consistent differences between two biological states (Increased expression vs. Decreased expression), Gene Set Enrichment Analysis (GSEA) was performed by the JAVA program (http://software.broadinstitute.org/gsea/downloads.jsp) using the MSigDB C2 KEGG pathways gene sets, which contains 186 gene sets. Normalized enrichment score (NES), nominal p value and false discovery rate (FDR) were used to quantify enrichment magnitude and statistical significance, respectively [16].

### Statistical analysis

Data analysis was performed with SPSS (Version 22.0; IBM, New York, NY, USA). Differences between groups were calculated by using Chi square test or Fisher exact test. Box-plots were used to visualize expression differences for Wnt ligands between tumor and adjacent non-tumor tissues. The optimal cut-off values for Wnt ligands expression were determined by X-tile software (Version3.6.1, Yale University, New Haven, CT, USA) [17]. Subsequently, each expression level of Wnt ligands was divided into increased expression group and decreased expression group according to the optimal cut-off value. Chi square test was also applied to evaluate the association between Wnt ligands expression and the clinicopathologic features in HCC. The Kaplan–Meier survival analysis and log-rank test were used to compare differences in survival times. Univariate and multivariate survival analysis were performed using the Cox hazards regression model to analyze the independent parameters associated to the overall survival and disease-free survival of HCC patients. A P value less than 0.05 was considered to be statistically significant.

## Results

### Basic patient characteristics

Clinical information and Wnt ligands mRNA expression levels of 360 patients were obtained from TCGA. Among 360 patients, 310 patients were recorded with diseases free survival status. Detailed clinical characteristics of the 360 patients in the TCGA database are shown in Table 1. TNM stage was significantly associated with the OS and DFS (P < 0.001), but not sex, age, BMI, or race (all P > 0.05).

**Table 1:** Basic characteristics of TCGA HCC patients

Clinical features	Overall survival					Disease-free survival				
	Patients (n = 360)	No.of events (%)	MST (months)	HR (95% CI)	P value	Patients (n = 310)	No. of events (%)	MST (month)	HR (95% CI)	P value
Age (year)										
< 60	165	54 (32.7%)	26.96	1.18 (0.83–1.68)	0.36	145	78 (53.8%)	20.54	0.78 (0.74–1.36)	0.98
≥ 60	195	75 (38.4%)	26.39			165	92 (55.7%)	19.75		
Sex										
Male	243	79 (32.5%)	25.94	1.18 (0.82–1.69)	0.38	203	114 (56.1%)	20.29	0.84 (0.64–1.22)	0.45
Female	117	50 (42.7%)	28.12			97	56 (57.7%)	19.74		
Race										
Asian	155	44 (28.3%)	25.87	0.75 (0.52–1.09)	0.13	138	69 (50.0%)	21.26	0.80 (0.59–1.09)	0.16
White + others	195	80 (41.0%)	27.51			165	98 (59.4%)	19.37		
Missing	10					7				
BMI										
≤ 25	174	62 (35.6%)	25.30	0.74 (0.51–1.06)	0.10	149	80 (53.7%)	18.50	0.85 (0.62–1.18)	0.33
> 25	153	49 (32.0%)	29.63			135	75 (55.5%)	23.09		
Missing	33					26				
Grade										
1–2	224	78 (34.8%)	27.13	1.14 (0.78–1.67)	0.49	192	101 (52.6%)	20.22	1.17 (0.85–1.61)	0.33
3–4	131	47 (35.8%)	25.57			114	65 (57.1%)	19.99		
Missing	5					4				
TNM stage										
I–II	248	67 (27.0%)	28.34	2.48 (1.71–3.62)	*<0.001*	215	101 (46.9%)	22.41	2.41 (1.72–3.36)	*<0.001*
III–IV	88	48 (54.5%)	22.54			74	55 (74.3%)	14.58		
Missing	24					21				

*TCGA* The Cancer Genome Atlas, *HCC* hepatocellular carcinoma, *BMI* body mass index, *TNM stage* tumor, node, metastasis stage

Italic values represent statistical significance

### Wnt ligands expression changes in HCC

All 19 members of the Wnt ligands family (Wnt1-Wnt16) had their expression analyzed in 360 HCC tumor tissues and 50 adjacent non-tumor tissues. The expression levels of Wnt2B, Wnt3A, Wnt6, Wnt8B and Wnt10B were significantly higher in primary liver tumor tissues than which in adjacent non-tumor tissues. However, the expression levels of Wnt2, Wnt5B, Wnt7A, Wnt7B, Wnt9A and Wnt11 were dramatically decreased in liver tumor tissues. Expression levels the remaining Wnt ligands, including Wnt1, Wnt3, Wnt4, Wnt5A, Wnt8A, Wnt9B, Wnt10A and Wnt16, remained insignificantly different between liver tumors tissues and adjacent non-tumor tissues. The box diagrams indicating the distribution of gene expression in HCC patients and adjacent normal for all Wnt ligands members are displayed in Additional file 1: Figure S1.

### Multivariate analysis and survival outcomes

**Fig. 1:**
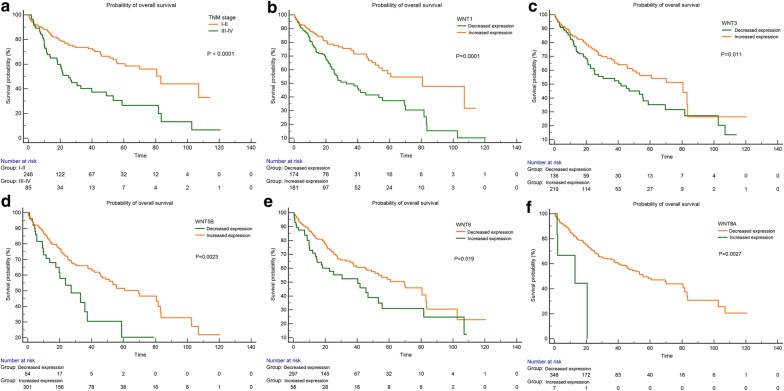
TMN stage (**a**) and expression of Wnt1, Wnt3, Wnt5B, Wnt6 and Wnt8A are associated with overall survival (**b**–**f**). Kaplan–Meier survival analysis and log-rank test were used to compare differences in overall survival between the groups classified using cut-off values determined by X-tile

The expression levels of Wnt ligands were divided into increased expression group and decreased expression group according to the cut-off values determined by X-tile program. The univariate and multivariate analysis demonstrated that TNM stages (HR = 2.02, 95% CI 1.37–2.96, P < 0.001), decreased Wnt1 expression (HR = 0.58, 95% CI 0.39–0.85, P = 0.006), decreased Wnt3 expression (HR = 0.65, 95% CI 0.45–0.96, P = 0.030), decreased Wnt5B expression (HR = 0.56, 95% CI 0.34–0.93, P = 0.023), increased Wnt6 expression (HR = 1.62, 95% CI 1.05–2.51, P = 0.030) and increased Wnt8A expression (HR = 4.14, 95% CI 1.47–11.68, P = 0.007) were independent prognostic factors for overall survival (Table 2) and TNM stages (HR = 1.41, 95% CI 1.24–1.76, P < 0.001), decreased Wnt1 expression (HR = 0.52, 95% CI 0.35–0.75, P < 0.001), increased Wnt3 expression (HR = 1.93, 95% CI 1.24–2.98, P = 0.003), decreased Wnt5A expression (HR = 0.61, 95% CI 0.40–0.91, P = 0.017), decreased Wnt5B expression (HR = 0.59, 95% CI 0.39–0.89, P = 0.011) and increased Wnt8B expression (HR = 0.53, 95% CI 0.36–0.77, P = 0.001) were independent prognostic factors for disease-free survival (Additional file 2: Table S1). Consistent with multivariate analysis, Kaplan–Meier survival analysis showed that HCC patients with advanced TNM stage, decreased Wnt1 expression, decreased Wnt3 expression, decreased Wnt5B expression, increased Wnt6 expression and increased Wnt8A expression had a worse OS than the counterpart (all P < 0.05) (Fig. 1). Kaplan–Meier survival analysis for DFS was shown in Additional file 3: Figure S2).

**Table 2:** Univariate and multivariate analysis of overall survival using the Cox proportional hazard regression model

Variables	Category	N	Univariate analysis		Multivariate analysis	
			HR (95% CI)	*p* Value	HR (95% CI)	*p*-Value
Age	< 60	165	1.18 (0.83–1.68)	0.36		
	≥ 60	195				
Sex	Male	243	1.18 (0.82–1.69)	0.38		
	Female	117				
Race	Asian	155	0.75 (0.52–1.09)	0.13		
	White + others	195				
BMI	≤25	174	0.74 (0.51–1.06)	0.10		
	>25	153				
Grade	1–2	224	1.14 (0.78–1.67)	0.49		
	3–4	131				
TNM stage	I–II	248	2.48 (1.71–3.62)	*< 0.001*	2.02 (1.37–2.96)	*< 0.001*
	III–IV	88				
Wnt1	Decreased (0–0.87)	175	0.50 (0.35–0.72)	*< 0.001*	0.58 (0.39–0.85)	*0.006*
	Increased (> 0.87)	185				
Wnt2	Decreased (0–1.62)	196	1.42 (1.00–2.00)	0.05		
	Increased (> 1.66)	164				
Wnt2B	Decreased (0–4.12)	188	1.29 (0.91–1.82)	0.19		
	Increased (> 4.12)	172				
Wnt3	Decreased (2.58–37.97)	140	0.64 (0.45–0.91)	*0.01*	0.65 (0.45–0.96)	*0.03*
	Increased (> 37.97)	220				
Wnt3A	Decreased (0–3.51)	258	1.32 (0.91–1.90)	0.15		
	Increased (> 3.51)	102				
Wnt4	Decreased (0–8.11)	99	1.49 (0.97–2.30)	0.07		
	Increased (> 8.11)	261				
Wnt5A	Decreased (0–416.79)	301	1.38 (0.87–2.16)	0.17		
	Increased (> 416.79)	59				
Wnt5B	Decreased (0.99–15.15)	58	0.51 (0.32–0.79)	*0.003*	0.56 (0.34–0.93)	*0.023*
	Increased (> 15.15)	302				
Wnt6	Decreased (0–9.01)	302	1.61 (1.08–2.41)	*0.02*	1.62 (1.05–2.51)	*0.03*
	Increased (> 9.01)	58				
Wnt7A	Decreased (0–0.24)	216	1.28 (0.90–1.82)	0.17		
	Increased (> 0.24)	144				
Wnt7B	Decreased (0–4.01)	239	1.42 (0.99–2.02)	0.05		
	Increased (> 4.01)	121				
Wnt8A	Decreased (0)	351	4.14 (1.51–11.3)	*0.006*	4.14 (1.47–11.68)	*0.007*
	Increased (> 0)	9				
Wnt8B	Decreased (0–2.17)	313	1.35 (0.84–2.15)	0.21		
	Increased (> 2.17)	47				
Wnt9A	Decreased (0–0.24)	183	1.34 (0.95–1.90)	0.10		
	Increased (> 0.24)	177				
Wnt9B	Decreased (0–2.19)	259	0.78 (0.52–1.16)	0.22		
	Increased (> 2.19)	101				
Wnt10A	Decreased (0–4.38)	186	0.99 (0.99–1.01)	0.80		
	Increased (4.38–173.47)	174				
Wnt10B	Decreased (0–6.16)	246	0.70 (0.47–1.03)	0.07		
	Increased (> 6.16)	114				
Wnt11	Decreased (0–28.88)	155	0.70 (0.49–0.99)	0.05		
	Increased (> 28.88)	205				
Wnt16	Decreased (0–1.62)	274	1.43 (0.97–2.13)	0.07		
	Increased (> 1.66)	86				

*BMI* body mass index, *TNM stage* tumor, node, metastasis stage

Italic values represent statistical significance

### Association with Wnt ligands expression and clinicopathologic variables

A total of 360 HCC patients with OS data and 310 HCC patients with DFS data were analyzed from TCGA. As showed in Table 3, low expression of Wnt1 and Wnt5B significantly correlated with the advanced TMN stage (Wnt1:P = 0.02; Wnt5B:P = 0.03). Low expression of Wnt3 correlated with histological grade (P = 0.04) but not TMN stage. Similar results were observed in the analysis for DFS. Low expressions of Wnt1 and Wnt5B correlated significantly with the advanced TMN stage (Wnt1:P = 0.02; Wnt5B:P = 0.02). Details were displayed in Additional file 4: Table S2).

**Table 3:** Correlation of clinicopathologic variables and expression of several specific Wnts significantly associated with OS

Clinical features	Patients (n = 360)	Wnt 1		P value	Wnt 3		P value	Wnt 5B		P value	Wnt 6		P value	Wnt 8A		P value
		Decreased	Increased		Decreased	Increased		Decreased	Increased		Decreased	Increased		Decreased	Increased	
Age (year)																
< 60	165	81	84	0.87	67	98	0.54	24	141	0.46	136	29	0.49	159	6	0.20
≥ 60	195	94	101		73	122		34	161		166	29		192	3	
Sex																
Male	243	124	119	0.19	98	145	0.42	41	202	0.57	204	39	0.96	237	6	0.95
Female	117	51	66		42	75		17	100		98	19		114	3	
Race																
Asian	155	77	78	0.64	64	91	0.46	29	126	0.22	130	25	0.94	153	2	0.17
White + others	195	92	103		73	122		27	168		163	32		188	7	
Missing	10															
BMI																
≤ 25	174	84	90	0.98	68	106	0.64	33	141	0.15	143	31	0.40	172	2	0.32
> 25	153	74	79		56	97		20	133		131	22		149	4	
Missing	33															
Grade																
1–2	224	107	117	0.53	78	146	*0.04*	37	187	0.75	183	41	0.19	221	3	0.06
3–4	131	67	64		60	71		20	111		114	17		125	6	
Missing	5															
TNM stage																
I–II	248	110	138	*0.02*	99	149	0.69	33	215	*0.03*	207	41	0.89	243	5	0.21
III–IV	88	52	36		33	55		20	68		74	14		84	4	
Missing	24															

*OS* overall survival, *BMI* body mass index, *TNM stage* tumor, node, metastasis stage

Italic values represent statistical significance

### GSEA identifies a Wnt1-related and Wnt5B-related KEGG signaling pathway

**Fig. 2:**
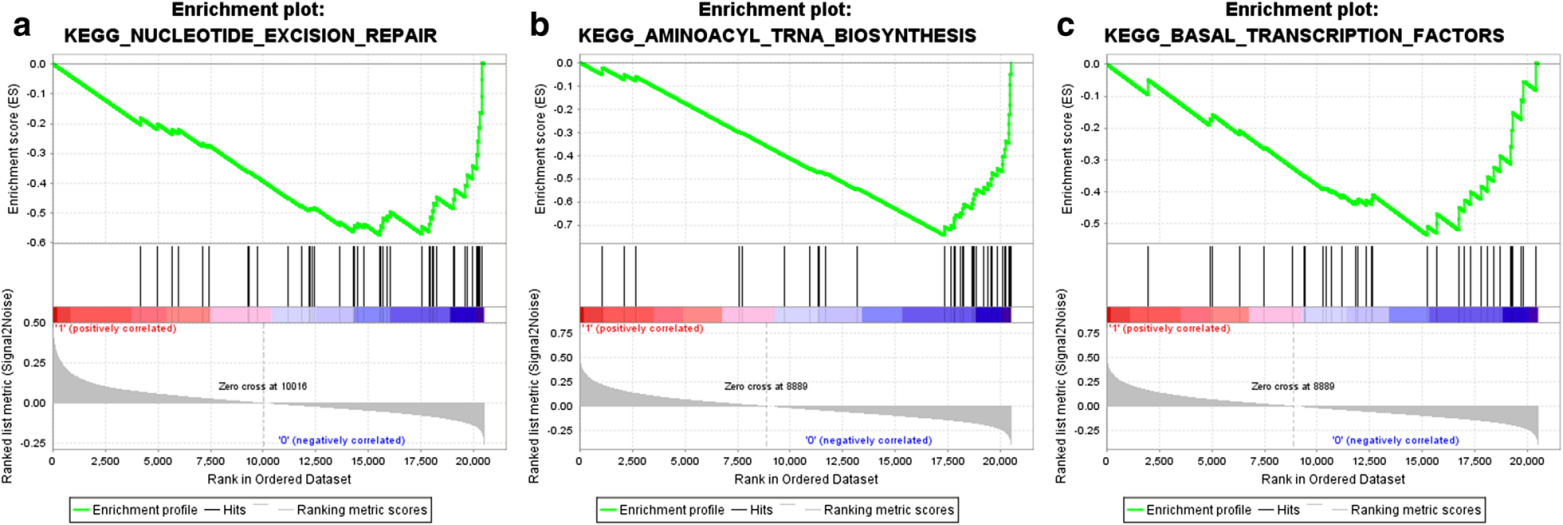
Enrichment plots from gene set enrichment analysis (GSEA). GSEA results showing nucleotide excision repair is differentially enriched in Wnt1 decreased expression phenotype (**a**) and aminoacyl tRNA biosynthesis (**b**) and basal transcription factors (**c**) are differentially enriched in Wnt5B decreased expression phenotype (**b**, **c**)

To identify KEGG signaling pathways that are differentially activated in HCC, we conducted GSEA between increased and decreased Wnt1 and Wnt5B expression data sets. GSEA reveal significant differences (NOM p-val < 0.01) in enrichment of MSigDB Collection (c2.cp.kegg.v6.1.entrez). We selected the most significantly enriched signaling pathways based on their normalized enrichment score (NES) (Fig. 2 and Table 4). The Fig. 2 shows that nucleotide excision repair is differentially enriched in Wnt1 low expression phenotype and aminoacyl tRNA biosynthesis and basal transcription factors are differentially enriched in Wnt5B low expression phenotype.

**Table 4:** Gene sets enriched in decreased expression phenotype

MSigDB collection	Gene	Gene set name	NES	NOM p-val	FDR q-val
c2.cp.kegg.v6.1.entrez.gmt	Wnt1	KEGG_NUCLEOTIDE_EXCISION_REPAIR	− 1.88	0.002	0.225
	Wnt5B	KEGG_AMINOACYL_TRNA_BIOSYNTHESIS	− 2.23	0.000	0.000
		KEGG_BASAL_TRANSCRIPTION_FACTORS	− 1.74	0.009	0.248

*NES* normalized enrichment score, *NOM* nominal, *FDR* false discovery rate. Gene sets with NOM p-val b0.05 and FDR q-val b0.25 are considered as significant

## Discussion

In this study, we investigated the association between Wnt ligand family genes and HCC. We observed that the mRNA expression levels of several specific Wnt ligand family genes, such asWnt1, Wnt3 and Wnt5B, are associated with distinct OS and DFS. Moreover, we found that expression of Wnt1 and Wnt5B significantly correlated with TMN stage. Thus, Wnt ligand family genes-especially Wnt1, Wnt3 and Wnt5B-may serve as prognostic biomarkers of HCC and represent possible oncogenes that could serve as therapeutic targets of HCC.

Wnt signals are known for regulation of diverse processes, including cell proliferation, survival, migration and polarization, embryonic development, specification of cell fate, and self-renewal in stem cells [18]. It is not surprising that Wnt pathway mutations are frequently observed in carcinomas. In the past decade, a large number of studies have been conducted to explore the role of Wnts and their downstream effectors in regulating cancer progression, including tumor initiation, tumor growth, cell senescence, cell death, differentiation and metastasis [18]. As activation of Wnt signals starts with the secretion of Wnt ligands, accumulating researches have investigated the expression profile of all 19 Wnt ligand genes indifferent cancer cell types, such as mammary carcinoma cell lines, human ovarian cancer cell lines and HCC cell lines [19–22]. Expectedly, Wnts and Wnt pathway components are frequently over- or under-expressed in different human malignant tumors. Interestingly, the expression patterns of Wnt signaling components can also serve as prognostic indicators of patient outcomes. For example, Wnt3a expression is reported to be significantly associated with poor prognosis of numerous cancers, including esophageal squamous cell carcinoma and HCC [10, 23]. In gastric cancer and ovarian carcinoma, increased level of Wnt5A protein was associated with high grade tumors and with decreased patient survival [24, 25], yet in colon cancer and HCC high level of Wnt5A protein correlated with increased patient survival [26, 27]. A recent study indicated that high Wnt2 expression in fibroblasts is associated with poor prognosis in human colorectal cancer [28]. However, a comprehensive analysis of the association between Wnts and liver cancer prognosis has not been performed.

In our study, it was found that increased expressions of Wnt 2B, Wnt3A, Wnt6, Wnt8B and Wnt10B in primary liver tumors and decreased expressions of Wnt2, Wnt5B, Wnt7A, Wnt7B, Wnt9A and Wnt11 in liver tumors tissues. These Wnts might have biomarker potential and could be utilized clinically in a diagnostic capacity. Interestingly, our study revealed that increased expressions of Wnt1, Wnt3, and Wnt5B and decreased expressions of Wnt6 and Wnt8A in HCC were associated with good OS probability. Increased expression of Wnt1, Wnt5A, Wnt5B and Wnt8B and decreased expression of Wnt3 were found to be associated with good DFS probability. The results showed that Wnt1, Wnt3 and Wnt5B were both independent prognostic factors affecting OS and DFS. Noteworthily, expression level of Wnt3 differed greatly for good OS and DFS probability. This reverse result could relate to the different cut-off values calculated by X-tile. As showed in Table 2 and Additional file 2: Table S1, the cut-off values of Wnt3 expression for OS and DFS were 37.97 and 98.6, respectively. Patients with low Wnt3 expression (< 37.97) had a worse OS and patients with low Wnt3 expression (< 98.6) had a better DFS. It could be explained that patients with Wnt3 expression level between 37.97 and 98.6 had a significantly better survival time and then contribute to this discrepancy.

Wnt1, one of the key ligands in β-catenin regulation, has been described for its prognostic role in several types of malignant tumors including non-small cell lung cancer [29], renal cell carcinoma [30], and colorectal cancer [31]. More importantly, a report by Lee et al. [11] found that high tumor Wnt1 expression was associated with increased hepatitis B virus (HBV)—related and hepatitis C virus (HCV)—related HCC recurrence after curative resection. The mechanism might correlate with increased nuclearβ-catenin accumulation accompanied by decreased membranous-cadherin expression and this seems to be consistent with the character of cancer cell metastasis. Surprisingly, our data showed that increased expression of Wnt1 was associated with good OS and DFS probability. The cause of this discrepancy between the previous study and our study remains unknown. It might be explained by the biological heterogeneity of HCC, which has an important impact on carcinogenesis and development.

Among various Wnts associated with the canonical Wnt/-catenin signaling pathway, Wnt3 was previously reported being frequently up-regulated in human cancers [8, 32, 33]. However, recent researches demonstrated that Wnt3 was down-regulated in some types of cancers and related to worse prognosis [34, 35]. With regards to HCC, Wnt3 expression was reported to be upregulated in human HCC compared to the adjacent peritumoral tissue [13]. In our study, the difference of Wnt3 expression remains insignificant between tumors tissue and adjacent non-tumor tissue. Moreover, since the cut-off values measured by X-tile differ too much for OS and DFS, the prognostic value of Wnt3 expression in HCC for a good OS and DFS was discrepant. Hence, prognostic value of Wnt3 expression in HCC remains further elucidation.

Wnt5B, an intermediately transforming or non-transforming Wnt family member, was reported to activate the noncanonical Wnt signals [36]. Several studies have demonstrated that Wnt5B is involved in the proliferation and migration of tumor cells and have critical role in tumor lymph angiogenesis and lymph node metastasis through the regulation of epithelial-mesenchymal transition (EMT) [37, 38]. Recently, an investigation from China reported that Wnt5b mRNA expression was significantly higher in hepatitis B virus-related HCC tissues than that of adjacent noncancerous tissues. Patients with up-regulated Wnt5b mRNA and protein had a shorter relapse-free survival [39]. However, our study indicated that increased expression of Wnt5b was significantly associated with better OS and DFS. Hence, similar to Wnt1, this discrepancy was observed in Wnt5B as well. We have a hypothesis that Wnt5B, who shows the greatest similarity with Wnt5A, may share a common Fzd receptor2 [40] and mediates similar Wnt5A effects of antagonizing Wnt signaling which may further inhibite HCC proliferation and migration [21, 37]. In addition, in our study, the optimal cut-off values for Wnt ligands expression were measured by X-tile, which presents substantial tumor subpopulations and shows the biological relationships between a biomarker and outcome. We believe the different methods to determine the cut-off value could also explain the discrepancy.

In recent years, a number of novel biomarkers, such as AFP [41, 42], glypican 3 [43], Osteopontin [44], CXCL1 [45], UQCRH [46], TIP30 [47], neutrophil–lymphocyte ratio [48], have emerged for diagnosing HCC and predicting patient outcome. Among these biomarkers, AFP is a well-known serum diagnostic and prognostic biomarker for HCC [49]. However, its prognostic value remains controversial. Several previous literature refuted the prognostic value of AFP in single, small HCC, and even for the prediction of HCC recurrence [50, 51]. Prognostic biomarkers’ use in the daily practice has never been endorsed by international guidelines. These frustrating results should not discourage the study of novel biomarkers and their translation at tissue level into prognostic and predictive indicators. In the present population, our work reveals a correlation between HCC outcome and Wnt ligand family genes. Hence, we postulate that the Wnt ligand family members may serve as potential serum biomarkers for prognosis of HCC.

Since the prognostic value of Wnt3 expression for OS and DFS is discrepant, we merely performed GSEA analysis to further investigate the potential mechanism of Wnt1 and Wnt5B in HCC. GSEA analysis showed that nucleotide excision repair is differentially enriched in Wnt1 low expression phenotype and aminoacyl tRNA biosynthesis and basal transcription factors are differentially enriched in Wnt5B low expression phenotype. Nucleotide excision repair (NER) is a repair system for many types of DNA damage, and therefore many types of genotoxic carcinogenic exposures, including ultraviolet light, products of organic combustion, metals, oxidative stress, etc. There have been a few reports demonstrating association of nucleotide excision repair with cancer [52, 53]. Ishikawa et al. [54] previously reported that the DNA repair system, especially the NER pathway, played a vital role in protection against human cancer. Moreover, Wang et al. [55] demonstrated that six polymorphisms of five genes involved in three steps of nucleotide excision repair pathways was associated with hepatocellular cancer risk. But the associations between Wnt1 and Wnt5B expression and nucleotide excision repair, aminoacyl tRNA biosynthesis and basal transcription factors were the first to be reported, and the regulatory mechanism needs to be further elucidated.

Nevertheless, the prediction of protein expression using mRNA was far from perfect. Because of limitations in our study design, the correlation between Wnts mRNA expression and Wnts protein expression could not be clearly assessed in this study. Further study in HCC is required.

## Conclusion

Our study identifies that several Wnt ligand family genes, such as Wnt1, Wnt3 and Wnt5B, may be potential prognostic biomarkers of HCC. Moreover, the nucleotide excision repair, aminoacyl tRNA biosynthesis and basal transcription factors may be the key pathway regulated by Wnts in HCC. Due to the small sample size and incomplete clinical information in this study, further experimental validation should be performed to prove the biologic impact of Wnts.

## Additional files

**Additional file 1: Figure S1.** Comparison of gene expression levels of all 19 members of Wnt family genes in tumor tissue and adjacent non-tumor tissue.

**Additional file 2: Table S1.** Univariate and multivariate analysis of disease-free survival using the Cox proportional hazard regression model.

**Additional file 3: Figure S2.** TMN stage (A) and expression of Wnt1, Wnt3, Wnt5A, Wnt5B and Wnt8B (B–F) are associated with disease-free survival. Kaplan–Meier survival analysis and log-rank test were used to compare differences in overall survival between the groups classified using cut-off values determined by X-tile.

**Additional file 4: Table S2.** Correlation of clinicopathologic variables and expression of several specific Wnts significantly associated with DFS.
