# Supplementary figures and images for: Expression of the Wnt ligands gene family and its relationship to prognosis in hepatocellular carcinoma
Source: Cancer Cell Int. 2019 Feb 15;19:34. doi: 10.1186/s12935-019-0743-z (PMC6376661; doi:10.1186/s12935-019-0743-z)

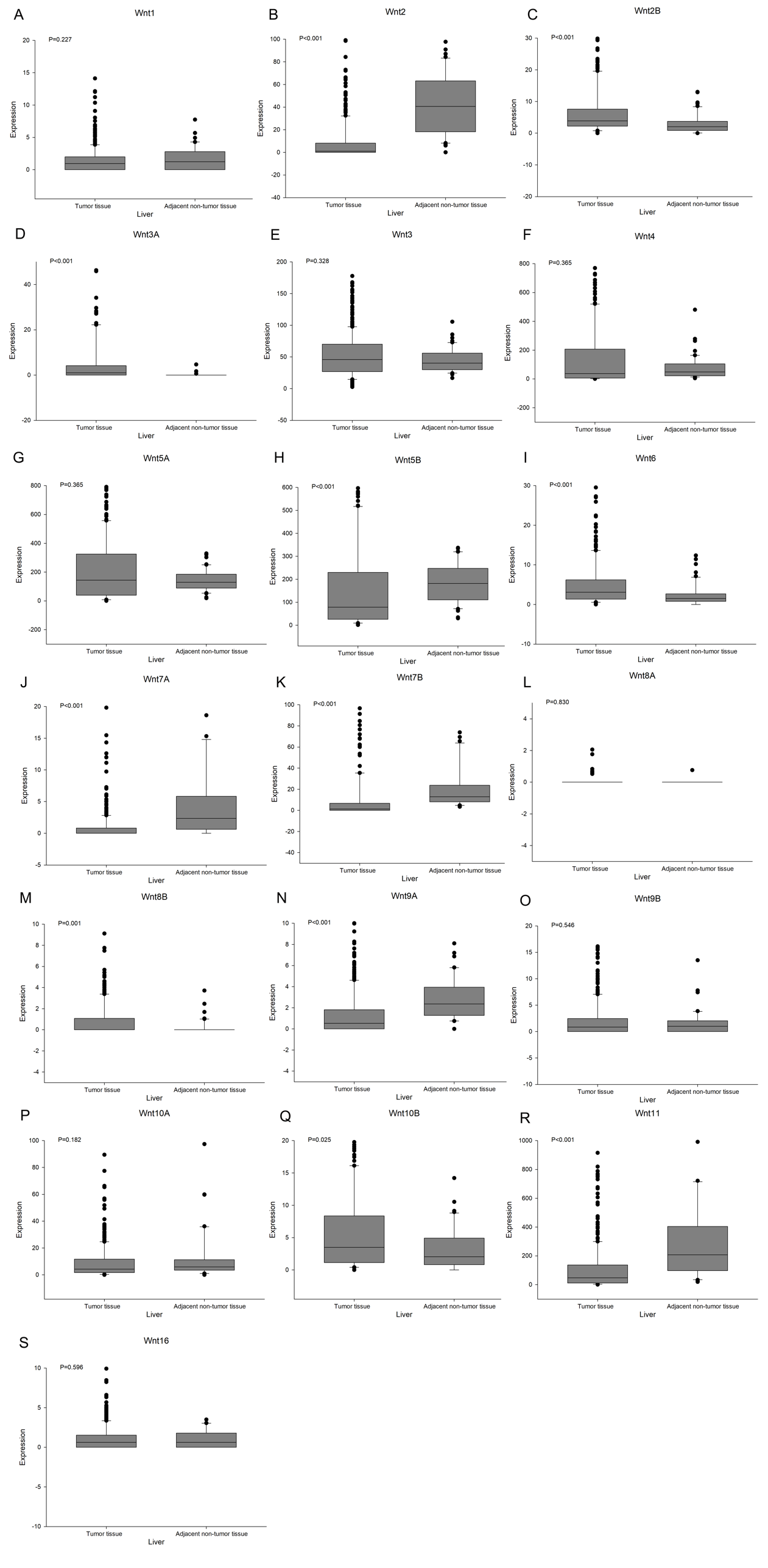

Supplement: Supplementary file 1 — Additional file 1: Figure S1. Comparison of gene expression levels of all 19 members of Wnt family genes in tumor tissue and adjacent non-tumor tissue. [file 12935_2019_743_MOESM1_ESM.tif]

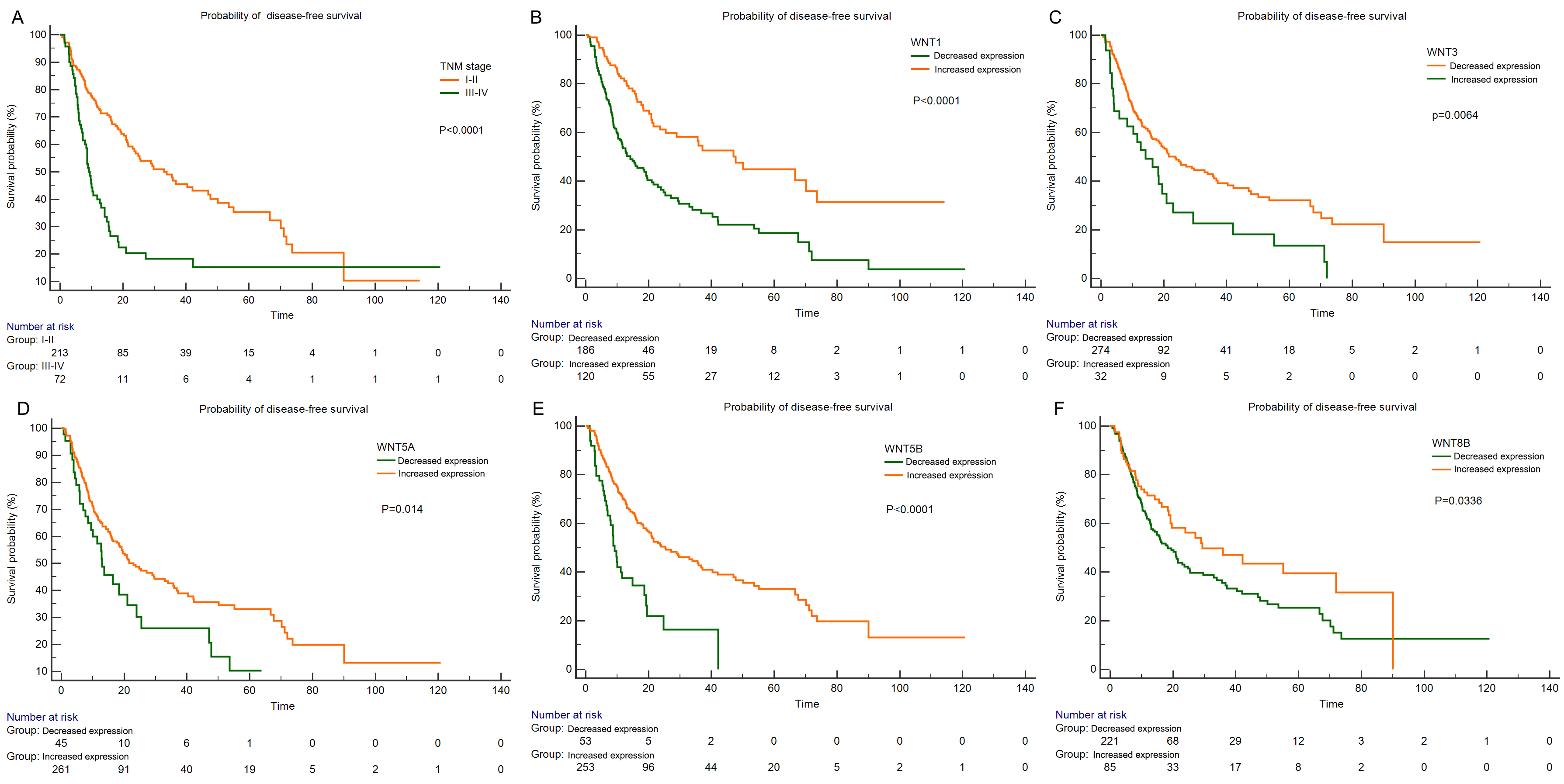

Supplement: Supplementary file 3 — Additional file 3: Figure S2. TMN stage (A) and expression of Wnt1, Wnt3, Wnt5A, Wnt5B and Wnt8B (B–F) are associated with disease-free survival. Kaplan–Meier survival analysis and log-rank test were used to compare differences in overall survival between the groups classified using cut-off values determined by X-tile. [file 12935_2019_743_MOESM3_ESM.tif]
