# Supplementary material for: Expression of the Wnt ligands gene family and its relationship to prognosis in hepatocellular carcinoma
Source: Cancer Cell Int. 2019 Feb 15;19:34. doi: 10.1186/s12935-019-0743-z (PMC6376661; doi:10.1186/s12935-019-0743-z)
Supplement: Supplementary file 2 — Additional file 2: Table S1. Univariate and multivariate analysis of disease-free survival using the Cox proportional hazard regression model. [file 12935_2019_743_MOESM2_ESM.docx]

Table S1 Univariate and multivariate analysis of disease-free survival using the Cox proportional hazard regression model.

| Variables | Category | N | Univariate analysis | | Multivariate analysis | |
| --- | --- | --- | --- | --- | --- | --- |
|  |  |  | HR (95% CI) | *p*-Value | HR (95% CI) | *p*-Value |
| Age | <60 | 165 | 0.78(0.74-1.36) | 0.98 |  |  |
|  | ≥60 | 195 |  |  |  |  |
| Sex | Male | 243 | 0.84(0.64-1.22) | 0.45 |  |  |
|  | Female | 117 |  |  |  |  |
| Race | Asian | 155 | 0.80(0.59-1.09) | 0.16 |  |  |
|  | White + others | 195 |  |  |  |  |
| BMI | ≤25 | 174 | 0.85 (0.62-1.18) | 0.33 |  |  |
|  | >25 | 153 |  |  |  |  |
| Grade | 1-2 | 224 | 1.17(0.85-1.61) | 0.33 |  |  |
|  | 3-4 | 131 |  |  |  |  |
| TNM stage | I-II | 248 | 2.41(1.72-3.36) | **<0.001** | 1.47(1.24-1.76) | **<0.001** |
|  | III-IV | 88 |  |  |  |  |
| Wnt1 | Decreased (0-1.26) | 188 | 0.43(0.31-0.60) | **<0.001** | 0.52(0.35-0.75) | **<0.001** |
|  | Increased (>1.26) | 122 |  |  |  |  |
| Wnt2 | Decreased (0-8.37) | 241 | 0.72(0.50-1.05) | 0.09 |  |  |
|  | Increased (>8.37) | 69 |  |  |  |  |
| Wnt2B | Decreased (0-3.26) | 130 | 0.77(0.57-1.04) | 0.08 |  |  |
|  | Increased (>3.26) | 180 |  |  |  |  |
| Wnt3 | Decreased (2.75-98.60) | 278 | 1.76(1.12-2.66) | **0.007** | 1.93(1.24-2.98) | **0.003** |
|  | Increased (>98.6) | 32 |  |  |  |  |
| Wnt3A | Decreased (0-2.02) | 200 | 0.65(0.46-0.90) | **0.01** |  |  |
|  | Increased (>2.02) | 110 |  |  |  |  |
| Wnt4 | Decreased (0-325.25) | 261 | 0.66(0.43-1.01) | 0.06 |  |  |
|  | Increased (>325.25) | 49 |  |  |  |  |
| Wnt5A | Decreased (0-13.20) | 46 | 0.61(0.41-0.91) | **0.015** | 0.61(0.40-0.91) | **0.017** |
|  | Increased (>13.20) | 264 |  |  |  |  |
| Wnt5B | Decreased (0.99-19.01) | 57 | 0.42(0.29-0.61) | **<0.001** | 0.59(0.39-0.89) | **0.011** |
|  | Increased (>19.01) | 253 |  |  |  |  |
| Wnt6 | Decreased (0-1.68) | 96 | 0.73(0.51-0.99) | **0.049** |  |  |
|  | Increased (>1.68) | 214 |  |  |  |  |
| Wnt7A | Decreased (0-0.62) | 218 | 0.62(0.43-0.89) | **0.009** |  |  |
|  | Increased (>0.62) | 92 |  |  |  |  |
| Wnt7B | Decreased (0-30.66) | 277 | 1.808(1.15-2.84) | **0.01** |  |  |
|  | Increased (>30.66) | 33 |  |  |  |  |
| Wnt8A | Decreased (0) | 7 | 1.38(0.34-5.60) | 0.65 |  |  |
|  | Increased (>0) | 303 |  |  |  |  |
| Wnt8B | Decreased (0-0.99) | 222 | 0.68(0.48-0.97) | **0.04** | 0.53(0.36-0.77) | **0.001** |
|  | Increased (>0.99) | 88 |  |  |  |  |
| Wnt9A | Decreased (0-1.02) | 224 | 1.19(0.86-1.66) | 0.28 |  |  |
|  | Increased (>1.02) | 86 |  |  |  |  |
| Wnt9B | Decreased (0-0.49) | 115 | 0.76(0.56-1.03) | 0.08 |  |  |
|  | Increased (>0.50) | 195 |  |  |  |  |
| Wnt10A | Decreased (0-7.41) | 205 | 1.00(0.99-1.01) | 0.98 |  |  |
|  | Increased (7.43-173.43) | 105 |  |  |  |  |
| Wnt10B | Decreased (0-16.11) | 278 | 1.53(0.97-2.40) | 0.06 |  |  |
|  | Increased (>16.11) | 32 |  |  |  |  |
| Wnt11 | Decreased (0-28.88) | 131 | 0.74(0.55-1.01) | 0.06 |  |  |
|  | Increased (>28.88) | 179 |  |  |  |  |
| Wnt16 | Decreased (0-3.06) | 272 | 1.36(0.86-2.15) | 0.19 |  |  |
|  | Increased (>3.06) | 38 |  |  |  |  |

Bold values represent statistical significance. Abbreviations: BMI: body mass index; TNM stage: tumor, node, metastasis stage.
