# Supplementary material for: Expression of the Wnt ligands gene family and its relationship to prognosis in hepatocellular carcinoma
Source: Cancer Cell Int. 2019 Feb 15;19:34. doi: 10.1186/s12935-019-0743-z (PMC6376661; doi:10.1186/s12935-019-0743-z)
Supplement: Supplementary file 4 — Additional file 4: Table S2. Correlation of clinicopathologic variables and expression of several specific Wnts significantly associated with DFS. [file 12935_2019_743_MOESM4_ESM.docx]

Table S2. Correlation of clinicopathologic variables and expression of several specific Wnts significantly associated with DFS.

| Clinical features | Patients  (n=310) | Wnt 1 | | P value | Wnt 3 | | P value | Wnt 5A | | P value | Wnt 5B | | P value | Wnt 8B | | P value |
| --- | --- | --- | --- | --- | --- | --- | --- | --- | --- | --- | --- | --- | --- | --- | --- | --- |
|  |  | Decreased | Increased |  | Decreased | Increased |  | Decreased | Increased |  | Decreased | Increased |  | Decreased | Increased |  |
| Age(year) |  |  |  |  |  |  |  |  |  |  |  |  |  |  |  |  |
| <60 | 145 | 93 | 52 | 0.24 | 130 | 15 | 0.99 | 24 | 121 | 0.43 | 22 | 123 | 0.17 | 105 | 40 | 0.77 |
| ≥60 | 165 | 95 | 70 |  | 148 | 17 |  | 22 | 143 |  | 35 | 130 |  | 117 | 48 |  |
| Sex |  |  |  |  |  |  |  |  |  |  |  |  |  |  |  |  |
| Male | 213 | 129 | 84 | 0.96 | 189 | 24 | 0.42 | 34 | 179 | 0.41 | 41 | 172 | 0.56 | 159 | 54 | 0.08 |
| Female | 97 | 59 | 38 |  | 89 | 8 |  | 12 | 85 |  | 16 | 81 |  | 63 | 34 |  |
| Race |  |  |  |  |  |  |  |  |  |  |  |  |  |  |  |  |
| Asian | 138 | 86 | 52 | 0.53 | 123 | 15 | 0.87 | 24 | 114 | 0.19 | 27 | 111 | 0.65 | 105 | 33 | 0.14 |
| White + others | 165 | 97 | 68 |  | 148 | 17 |  | 20 | 145 |  | 29 | 136 |  | 113 | 52 |  |
| Missing | 7 |  |  |  |  |  |  |  |  |  |  |  |  |  |  |  |
| BMI |  |  |  |  |  |  |  |  |  |  |  |  |  |  |  |  |
| ≤25 | 149 | 91 | 58 | 0.85 | 133 | 16 | 0.60 | 26 | 123 | 0.34 | 29 | 120 | 0.67 | 112 | 37 | 0.24 |
| >25 | 135 | 81 | 54 |  | 123 | 12 |  | 18 | 117 |  | 23 | 112 |  | 93 | 42 |  |
| Missing | 26 |  |  |  |  |  |  |  |  |  |  |  |  |  |  |  |
| Grade |  |  |  |  |  |  |  |  |  |  |  |  |  |  |  |  |
| 1-2 | 192 | 113 | 79 | 0.29 | 173 | 19 | 0.68 | 33 | 159 | 0.11 | 31 | 161 | 0.21 | 141 | 51 | 0.35 |
| 3-4 | 114 | 74 | 40 |  | 101 | 13 |  | 12 | 102 |  | 25 | 89 |  | 78 | 36 |  |
| Missing | 4 |  |  |  |  |  |  |  |  |  |  |  |  |  |  |  |
| TNM stage |  |  |  |  |  |  |  |  |  |  |  |  |  |  |  |  |
| I-II | 215 | 122 | 93 | **0.02** | 190 | 25 | 0.40 | 29 | 186 | 0.16 | 32 | 183 | **0.02** | 153 | 62 | 0.94 |
| III-IV | 74 | 53 | 21 |  | 68 | 6 |  | 15 | 59 |  | 20 | 54 |  | 53 | 21 |  |
| Missing | 21 |  |  |  |  |  |  |  |  |  |  |  |  |  |  |  |

Bold values represent statistical significance. Abbreviations: DFS: disease-free survival; BMI: body mass index; TNM stage: tumor, node, metastasis stage.
